# Single Cell Transcriptomics of Ependymal Cells Across Age, Region and Species Reveals Cilia-Related and Metal Ion Regulatory Roles as Major Conserved Ependymal Cell Functions

**DOI:** 10.3389/fncel.2021.703951

**Published:** 2021-07-15

**Authors:** Adam MacDonald, Brianna Lu, Maxime Caron, Nina Caporicci-Dinucci, Dale Hatrock, Kevin Petrecca, Guillaume Bourque, Jo Anne Stratton

**Affiliations:** ^1^Department of Neurology and Neurosurgery, Montreal Neurological Institute, McGill University, Montreal, QC, Canada; ^2^Department of Human Genetics, McGill University, Montreal, QC, Canada

**Keywords:** ependymal cell, ependyma, human, mouse, neonatal, adult, brain homeostasis, single-cell RNA-seq (scRNA-seq)

## Abstract

Ependymal cells are ciliated-epithelial glial cells that develop from radial glia along the surface of the ventricles of the brain and the spinal canal. They play a critical role in cerebrospinal fluid (CSF) homeostasis, brain metabolism, and the clearance of waste from the brain. These cells have been implicated in disease across the lifespan including developmental disorders, cancer, and neurodegenerative disease. Despite this, ependymal cells remain largely understudied. Using single-cell RNA sequencing data extracted from publicly available datasets, we make key findings regarding the remarkable conservation of ependymal cell gene signatures across age, region, and species. Through this unbiased analysis, we have discovered that one of the most overrepresented ependymal cell functions that we observed relates to a *critically* understudied role in metal ion homeostasis. Our analysis also revealed distinct subtypes and states of ependymal cells across regions and ages of the nervous system. For example, neonatal ependymal cells maintained a gene signature consistent with developmental processes such as determination of left/right symmetry; while adult ventricular ependymal cells, not spinal canal ependymal cells, appeared to express genes involved in regulating cellular transport and inflammation. Together, these findings highlight underappreciated functions of ependymal cells, which will be important to investigate in order to better understand these cells in health and disease.

## Introduction

Ependymal cells are ciliated glial cells that form an epithelial barrier, called the ependyma, lining the brain’s ventricular system and the spinal cord’s central canal. They develop from radial glia along the surface of the ventricles of the brain and spinal canal starting from the first postnatal days, thereby providing an interface between the parenchyma and cerebrospinal fluid (CSF)-filled cavities throughout life. This interface allows ependymal cells to control the bidirectional passage of immune cells and solutes between the CSF and interstitial fluid (Alvarez and Teale, 2007; Mastorakos and McGavern, 2019); while also providing homeostatic regulation of molecules (Bedussi et al., 2015; Ma et al., 2017); in addition to playing a critical role in sensing and propelling CSF *via* primary and motile cilia, respectively (Bolborea et al., 2015).

Three subtypes of ependymal cells have been described: E1, E2, and E3 ependymal cells. The three distinct ependymal cell subtypes are defined by their cilia number and regional distribution (Mirzadeh et al., 2008, 2017) where E1 ependymal cells possess multiple motile cilia and are the most abundant subtype in the adult brain, occupying the majority of the lateral (forebrain), third (midbrain) and fourth (hindbrain) ventricles. E2 cells possess both primary and motile cilia and are bi-ciliated (Mirzadeh et al., 2008) and line the spinal canal (Alfaro-Cervello et al., 2012) in addition to occupying a portion of the third and fourth ventricles of the brain (Mirzadeh et al., 2017); while E3 ependymal cells have primary cilia, are uniciliated (Mirzadeh et al., 2017), and exist primarily in a small portion of the third ventricle, inhabiting the preoptic and infundibular recesses (Mirzadeh et al., 2017). The multitude of functions and diverse roles that these cells play is understudied (Lorencova et al., 2020).

In this study, publicly available single-cell transcriptomic datasets were screened to identify datasets encompassing ependymal cells across age, nervous system region, and species. Following quality control (QC) scrutiny, we were able to identify four datasets encompassing high-quality ependymal cells, including *mouse* ependymal cells from the adult and neonatal forebrain, and the spinal cord, in addition to ependymal cells from the adult human forebrain. By comparing datasets, we find striking homogeneity across ependymal cells in age, species, and region. This included a highly conserved gene signature related to metal ion regulation functions, which we confirmed using RNAscope—a fluorescence *in situ* hybridization strategy. Yet we also identified gene signatures that distinguished ependymal cells from neonatal and adult contexts, in addition to gene signatures that distinguished between the brain and spinal cord regions, which suggests that ependymal cell states and subtypes also have differing functions and mechanisms for maintaining CNS health.

## Materials and Methods

### Single-Cell RNAseq Data Sourcing

Neonatal mouse forebrain dataset (*n* = 3) was accessed *via* GEO: GSE123335 (Loo et al., 2019), adult mouse forebrain dataset (*n* = 4) was accessed *via* GEO: GSE100320 (Shah et al., 2018), while the mouse spinal cord dataset (*n* = 3) was accessed from the PaglaoDB database under SRA/SRS accession numbers SRA667466/SRS3059989, SRA667466/SRS3059990, and SRA667466/SRS3059991. The human forebrain dataset (*n* = 5) was provided by Dr. Petrecca and can be made available by the corresponding author upon request.

### Single-Cell RNAseq Quality Control

All datasets were imported into the Seurat (v4.0.1) R toolkit for QC and downstream analysis (Macosko et al., 2015). All functions were run with default parameters unless specified otherwise. Cells were excluded during QC selection according to recommended Seurat parameters: if the number of unique genes detected was less than 200, if the number of unique genes detected was greater than 2,500, or if the percentage of mitochondrial genes expressed was greater than 5% (or 10% in the case of the adult brain datasets). Gene expression was log normalized to a scale factor of 10,000. Following QC cut-offs, we identified 18,357 unique features across 41 ependymal cells in the neonatal dataset, 15,259 unique features across 142 ependymal cells in the adult mouse brain dataset, 24,506 unique features across 344 ependymal cells in the spinal cord dataset, and 18,312 unique features across 33 ependymal cells in the adult human brain datasets.

### Single Cell Analysis

**Figure 1:**
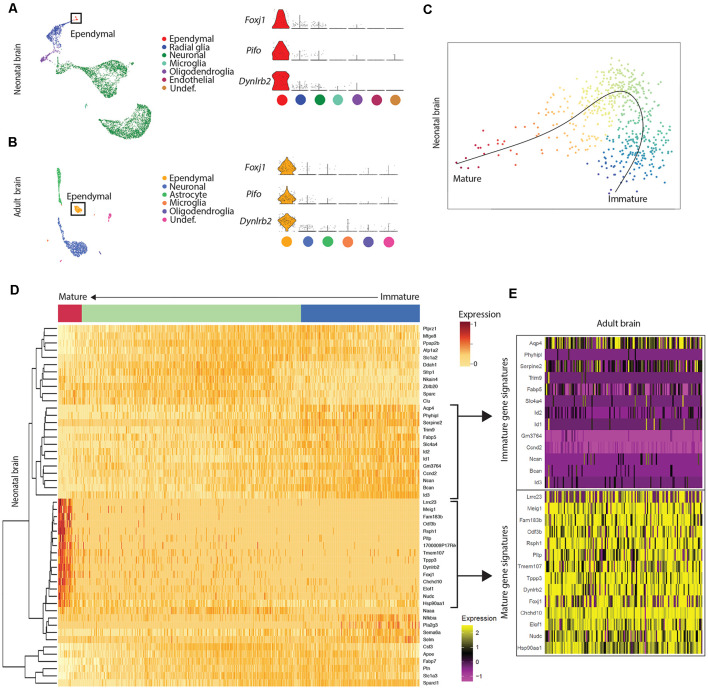
Mature ependymal cells are detectable in the brain by postnatal day 0 and are maintained in adulthood. **(A)** UMAP plot of single-cell transcriptional profiles from neonatal mouse brain. One cluster in the neonatal brain was identified as ependymal cells (box) as per the expression of ependymal cell marker genes such as *Foxj1*, *Pifo*, and *Dynlrb2* (presented in violin plots). **(B)** UMAP plot of single-cell transcriptional profiles from adult mouse brain. One cluster was identified as ependymal cells (box) as per the expression of ependymal cell marker genes such as *Foxj1*, *Pifo*, and *Dynlrb2* (presented in violin plots). **(C)** PCA plot depicting pseudotime trajectory of ependymal cell development in neonatal mouse brain. Ependymal cells (*Foxj1*+ cluster) and radial glia (*Gfap*+ cluster; ependymal cell precursor cells) were extracted from the neonatal dataset and then pseudotime ordering was performed using Slingshot. This revealed a curved trajectory (line) where radial glia (immature ependymal cells, blue) give rise to mature ependymal cells (red) *via* an ordered progression of transcriptional changes. X- and Y-axis represent the first and second raw component scores, respectively. **(D)** Heatmap of differentially expressed genes generated from pseudo-ordered data in **(C)**. This revealed the top 50 most dynamically expressed genes across the pseudo-ordered state (Mature, red; Intermediate, green; Immature, blue). **(E)** Heatmap of adult brain ependymal cells. Selected genes representative of immature ependymal cells (blue in **D**) and mature ependymal states (red in **D**) from the neonatal dataset were plotted demonstrating a striking overlap between mature genes in neonatal and adult ependymal cells. Note the lack of immature gene signatures in adult ependymal cells is consistent with the adult ependymal cell population being fully mature by this stage.

All codes are available at Github^1^. Pseudotime was performed using the Slingshot (v1.8.0) package (Street et al., 2018) where analysis was conducted on ependymal cell and radial glia clusters identified by the expression of marker genes *Foxj1* and *Gfap*, respectively. Default parameters were used throughout the workflow. An associated heatmap was generated as part of the standard Slingshot workflow. GO-term analysis was performed using the clusterProfiler (v3.18.1) package (Yu et al., 2012) where the top 200 differentially expressed genes were input (significantly regulated; *p*-value < 0.05 following Bonferonni correction), and the output figure contained the top 30 most significant GO-terms. *P*-values were adjusted by false discovery rate during clusterProfiler analysis. clusterProfiler (v3.18.1) was also used to generate the cnetplots provided in Supplemental Material, using the top 30 GO-terms, top 200 genes, and otherwise default parameters. Unless otherwise stated, heatmaps were generated using default parameters for the DoHeatmap function in Seurat. For heatmap in Figure 1E: immature and mature genes from neonatal pseudotime analysis were input and the heatmap was generated using the adult dataset. Technical limitations prevented us from including one gene (1700009P17Rik) as it was not found in the adult dataset. For heatmap in Figure 5A: ependymal cells from each mouse dataset were integrated using SCTransform and the top 60 differentially expressed genes were determined (significantly regulated; *p*-value < 0.05 following Bonferonni correction) by comparing genes up in neonatal ependymal cells, genes up in adult ependymal cells, genes up in spinal cord ependymal cells, genes down in neonatal ependymal cells, genes down in adult ependymal cells, and genes down in spinal cord ependymal cells compared to all other ependymal cells in the merged dataset. Heatmap was created with parameters adjusted to include the top 10 significant genes for each comparison.

**Figure 2:**
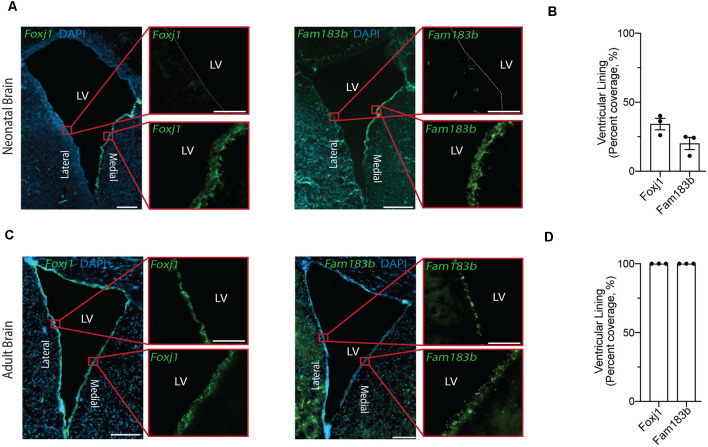
*In situ* detection of mature ependymal cell genes in the ventricular lining of neonatal and adult brain validates the presence of mature ependymal cells from postnatal day 0 onwards. **(A,C)** Representative images of neonatal **(A)** and adult **(C)** mouse brains demonstrate the expression of *Foxj1* (left panels) and *Fam183b* (right panels) along the ventricles at both ages (green). Note the localization of mature ependymal cells along the medial wall of the neonatal ventricle suggesting the temporal progression of ependymal cell development follows a medial to lateral dynamic. LV = Lateral Ventricle. Scale bars: 200 μm (**A,C** overview); 50 μm (**A,C** zoomed). Dotted lines show ventricle-ependyma interface. **(B,D)** Quantification of the percentage of cells (DAPI+) expressing *Foxj1* or *Fam183b* along the ventricular lining of the neonatal **(B)** and adult **(D)** mouse brain (*n* = 3; mean ± SEM).

**Figure 3:**
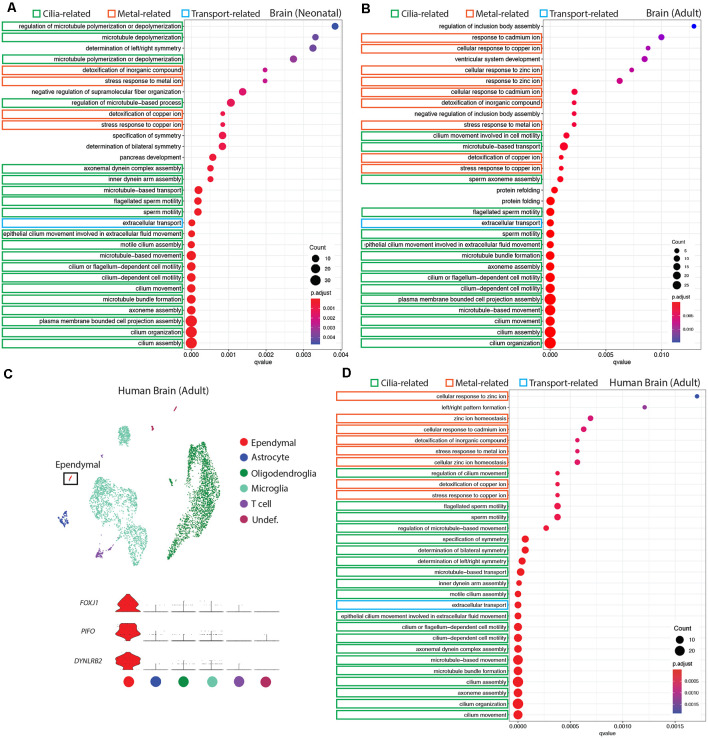
Conservation of ependymal cell GO-terms in the ventricular lining of the brain across age and species. **(A,B,D)** Dot plots representing GO-terms generated from the top 200 differentially expressed genes in ependymal cells (vs. all other cell types in the respective dataset) were generated using clusterProfiler. The top 30 most significant GO-terms were plotted. GO-terms clustered in three major categories that were shared across age and species: cilia-related terms (green), metal ion-related terms (orange), and transport-related terms (blue). Dot size represents gene counts in the respective pathway. Node color intensity shows enrichment degree (*p*-values adjusted by FDR). *Q*-values were assigned to the horizontal axis to control for false positive discovery rate. **(A)** GO-terms in the neonatal mouse brain (20/30 terms are cilia-related, 4/30 are metal ion-related, 1/30 is transport-related, and 5/30 were other functions unique to the neonatal dataset). **(B)** GO-terms in the adult mouse brain (15/30 terms are cilia-related, 9/30 terms are metal ion-related, 1/30 is transport-related, and 5/30 were other functions unique to the adult dataset). **(C)** UMAP plot of single-cell transcriptional profiles from the adult human brain. One cluster was identified as ependymal cells (box) as per the expression of ependymal cell marker genes such as *Foxj1*, *Pifo*, and *Dynlrb2* (presented in violin plots). **(D)** GO-terms in the adult human brain (20/30 functions are cilia-related, 8/30 terms are metal ion-related, 1/30 is transport-related, and 1/30 is related to a miscellaneous function).

**Figure 4:**
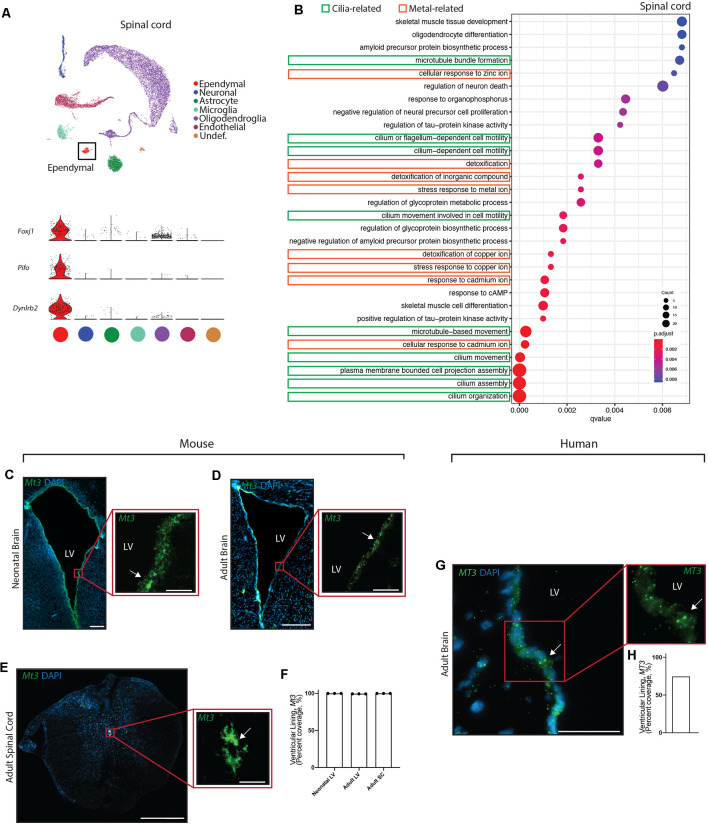
Conservation of ependymal cell GO-terms and genes related to metal ion regulatory functions across age, species, and region. **(A)** UMAP plot of single-cell transcriptional profiles from adult mouse spinal cord. One cluster was identified as ependymal cells (box) as per the expression of ependymal cell marker genes such as *Foxj1*, *Pifo*, and *Dynlrb2* (presented in violin plots). **(B)** Dot plots representing GO-terms generated from the top 200 differentially expressed genes in ependymal cells (vs. all other cell types in the respective dataset) were generated from the adult mouse spinal cord dataset. The top 30 most significant GO-terms were plotted. GO-terms clustered in two major categories: cilia-related terms (green) and metal ion-related terms (orange; 9/30 terms are cilia-related, 8/30 terms are metal ion-related, and 13/30 were other functions unique to the spinal cord dataset). Dot size represents gene counts. Node color intensity shows enrichment degree (*p*-values adjusted by FDR). *Q*-values were assigned to the horizontal axis to control for false positive discovery rate. **(C–E)** Representative images of the neonatal ventricular lining **(C)**, adult ventricular lining **(D)**, and spinal canal **(E)** demonstrates the expression of a gene associated with metal ion buffering (*Mt3*) in mouse tissue (arrow). **(F)** Quantification of the percentage of cells (DAPI+) expressing *Mt3* along the ventricular and canal linings in mouse brain and spinal cord (*n* = 3; mean ± SEM). Note the localization of *Mt3* along the entire lining, including in the neonatal brain along the lateral border where mature ependymal cells do not reside. This suggests that metal ion related functions of the ventricular lining may be initiating even before the full maturation of ependymal cells. **(G)** Representative image of the adult human brain demonstrates the expression of *MT3* in the ventricular lining (arrow). **(H)** Quantification of the percentage of cells (DAPI+) expressing *MT3* along the ventricular lining in human brain. Scale bars: 200 μm (**C–E** overview), 50 μm (**C–E** zoomed; and **G**). LV = Lateral Ventricle.

**Figure 5:**
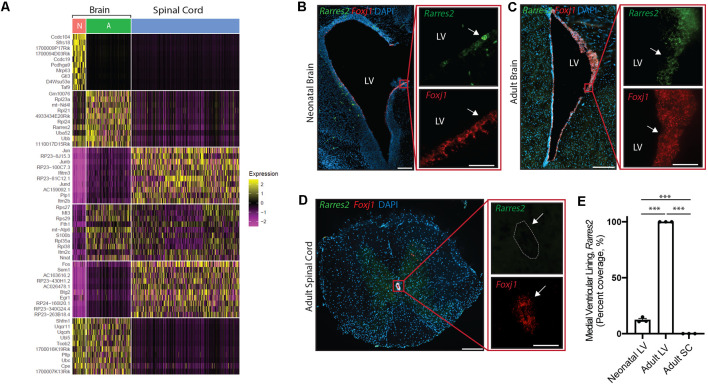
Unique ependymal cell signatures across age and region. **(A)** Heatmap displaying the top 60 differentially expressed ependymal cell genes from the neonatal mouse brain (N, red), the adult mouse brain (A, green), and the mouse spinal cord (blue). **(B–D)** Representative images demonstrating expression of *Rarres2* (green) and *Foxj1* (red) in ependymal cells (arrow) of the neonatal mouse brain **(B)**, adult mouse brain **(C)**, and the mouse spinal cord **(D)**. Note the complete lack of expression of *Rarres2* in ependymal cells in the spinal canal as well as only a subset of cells expressing *Rarres2* in the ventricular lining in early development compared to 100% expression in the adult ventricular lining. Scale bars: 200 μm (**B–D** overview); 50 μm (**B–D** zoomed). **(E)** Quantification of the percentage of cells (DAPI+) expressing *Rarres2* along the ventricular (medial) and spinal canal lining (*n* = 3; mean ± SEM). ***p < 0.0001. In **(D)**, dotted lines show the central canal-ependymal lining interface.

### Tissue

C57B6/J mice were used (Charles River Laboratories). Neonatal animals were postnatal day 0–1 (*n* = 3 per group), while adult animals were 12 weeks old (*n* = 3 per group). Mice were housed in a vivarium under pathogen-free conditions in cages of up to five animals. Studies were approved by the McGill University Animal Care and Use Committee (UACC) under animal use protocol 2019-8102. UACC guidelines on the ethical use and care of animals were followed. Human brain autopsy tissue was provided by Dr. Prat (U of Montreal) under McGill University Health Centre REB ethics #2020-6185.

### *In situ* Hybridization

Following cardiac 4% PFA perfusion, brains were harvested and post-fixed in PFA for 2 h. Brains were then kept in 30% sucrose at 4°C until they sank (48 h for mature brain, 24 h for neonatal brain), at which point brains were individually snap-frozen in O.C.T. (Tissue-Tek) and stored at −80°C. Tissue was coronally cryosectioned at 15 μm on Superfrost Plus slides (Fisher) at −20°C and stored at −80°C until processing for RNAscope (Advanced Cell Diagnostics, ACD). Human tissue was freshly collected then snap-frozen in OCT. Following cryosectioning, human tissue was fixed on-slide in 4% PFA for 15 min. For all tissue, RNA *in situ* hybridization was performed using RNAscope^®^ (ACD). Tissue was processed according to the protocol set out in the user manual for fixed-frozen tissue unless otherwise stated. Briefly, slides were incubated in a target retrieval solution at 95°C for 15 min. Sections were then washed with 100% EtOH and air-dried for 5 min at room temperature. Sections were ringed using hydrophobic barrier pen (ImmEdge, Vector Labs; Cat. No. 310018) and then incubated with protease III for 20 min at room temperature. Sections were then washed once with PBS before carrying out RNAscope multiplex assays according to the manufacturer’s protocols, albeit we used one single wash following each incubation to minimize tissue erosion. All incubations were at 40°C and used a humidity control chamber (HybEZ oven, ACD; Cat. No. 321711). Mouse probes were: Foxj1-C2 (Cat. No. 317091-C2), Fam183b (Cat. No. 515951), Mt3 (Cat. No. 504061), and Rarres2 (Cat. No. 572581), in addition to one human probe MT3-C2 (Cat. No. 525421-C2). Opal dye 520 and 570 fluorophores (Cat. No. FP1487001KT and Cat. No. FP1488001KT, respectively; Akoya Biosciences) were diluted in RNAscope TSA dilution buffer (1:1,000; Cat. No. 322809). Nuclei were labeled with DAPI (1/5,000; Invitrogen). Slides were mounted in PermaFluor Mounting Media (Thermofisher). Sections were imaged using a Zeiss Axio Observer (20× tile scan for the overview, 40× for zoomed images).

FIJI (ImageJ) was used to measure ventricle lining length and to measure the length of probe coverage along lining to calculate a percentage value that represents the coverage over the ventricle or canal. For *Rarres2* quantification, we measured the length of probe coverage along the medial ventricular lining. Statistical analyses were conducted with Graphpad Prism 8 and significance levels were obtained using one-way ANOVAs using Holm–Sidak’s test to correct for multiple comparisons.

## Results

Publicly available single-cell transcriptomic datasets were screened to identify datasets encompassing ependymal cells across age, region, and species. We extensively screened the literature and single-cell databases (PanglaoDB, Single Cell Portal, Single Cell Expression Atlas, etc.) to identify ependymal cell populations from diverse environments and conditions. Following QC scrutiny, we were able to identify four datasets encompassing high-quality ependymal cells, including ependymal cells from adult and neonatal forebrain, and from spinal cord in the mouse, in addition to ependymal cells from the adult human brain.

### Mature Ependymal Cells Are Detectable in the Brain by Postnatal Day 0 and Are Maintained in Adulthood

Our goal was to compare ependymal cells at their earliest stage of maturity with ependymal cells that were well established mature cells in the adult forebrain. By comparing ependymal cells at two polar ages where the microenvironments are highly unique, we hypothesized that any similarities would likely reflect the most conserved and relevant gene signatures underlying critical ependymal cell-related functions for the maintenance of the brain. As such, we first sourced a single cell dataset at postnatal day 0, an age that others have shown to be one of the earliest stages of mature ependymal cell generation in the forebrain (Harkins et al., 2021). Indeed, our clustering analysis in postnatal day 0 brain identified one defined cluster expressing genes that were highly specific to mature ependymal cells (Figure 1A). This included genes such as: *Foxj1*, a transcription factor which acts as a master regulator of ciliogenesis (Jacquet et al., 2009); *Pifo*, a protein-coding gene involved in ciliary movement (Kinzel et al., 2010); and *Dynlrb1*, a protein-coding gene involved in dynein-mediated transport (Terenzio et al., 2020). We similarly identified this same ependymal cell population in an adult brain dataset (Figure 1B). To establish whether mature cells from postnatal day 0 had an extensive gene signature reflective of a mature ependymal cell state, we defined the most mature gene signatures detectable in neonatal ependymal cells and compared this to adult ependymal cells. To accomplish this, we performed pseudotime ordering of ependymal cells as well as their precursor cells (radial glia) which were also detectable in the postnatal day 0 mouse brain (Figure 1C). This approach allowed us to order cells based on their developmental progression and identify gene signatures reflective of both the most mature and immature states (Figure 1D). Strikingly, 14/15 genes most associated with the mature state in the postnatal day 0 brain (including *Lrcc1, Meig1, Fam183b, Foxj1* etc.) were also highly expressed in adult ependymal cells suggesting that fully mature ependymal cells are detectable at postnatal day 0 (Figure 1E). Similarly, the majority of genes that were characteristic of the immature state (*Phyhipl, Ncan, Bcan* etc.) were not expressed by adult ependymal cells, consistent with the adult ependymal cell population being fully mature in adulthood. All further bioinformatic analysis was completed on the mature ependymal cell cluster from the neonatal and adult brain. We also performed validation experiments using RNAscope technology (Wang et al., 2012) on tissue sections collected from neonatal mice and postnatal week 12 adult mice (*n* = 3 per group). The two genes we assessed, which are associated with the mature state (*Foxj1* and *Fam183b*) were expressed in the ventricular lining at both ages (Figures 2A–D). Albeit the entire ventricular lining of the adult brain expressed *Foxj1* and *Fam183b*, only a portion of the lining of the neonatal brain expressed these markers (*Foxj1*: 34.22% ± 4.18%; *Fam183b*: 20.12% ± 4.45). This expression in the neonatal lining was restricted to the medial wall suggesting that the temporal progression of ependymal cell development follows a medial to lateral dynamic as others have described (Abdelhamed et al., 2018).

### Conservation of a Homogenous Ependymal Cell Transcriptome Across Age, Species, and Region

It is well established that E1 ependymal cells monopolize the lateral ventricles of the forebrain and are the major ependymal cell subtype in the brain (Ohata and Alvarez-Buylla, 2016). In order to determine how this ependymal cell subtype is affected by age and species, we used gene ontology (GO)-term analysis to compare *mouse* neonatal and adult ependymal cells (from datasets described above), as well as adult *human* ependymal cells, all derived from the forebrain (Figures 3A,B,D, Supplementary Figure 1). As with *mouse* neonatal and adult datasets, clustering analysis was performed on adult *human* brain tissue in order to identify any cluster expressing genes that were specific to mature ependymal cells in human tissue. Indeed, we found one cluster expressing *Foxj1*, *Pifo*, and *Dynlrb1* (Figure 3C). All ependymal cell clusters across datasets were compared to all other cell types in their respective datasets to generate the top 200 differentially expressed ependymal cell genes (for all genes significantly upregulated, *p* < 0.001 following Bonferroni correction, see Supplementary Table 1) then clusterProfiler was used to generate the top 30 most significant GO-terms for each dataset. GO-terms clustered in three major categories that were shared across age and species, including cilia-related terms, metal ion-related terms, and transport-related terms. Strikingly, GO-terms associated with metal ion-related functions made up 4/30, 9/30, and 8/30 of GO-terms in *mouse* neonatal, *mouse* adult, and *human* adult datasets, respectively, demonstrating conservation of this *critically* understudied function for ependymal cells (Zatta et al., 2008). Not surprisingly 20/30, 15/30, and 20/30 of GO-terms in *mouse* neonatal, *mouse* adult, and *human* adult datasets, respectively, were related to cilia consistent with the field focusing largely on this aspect of ependymal cell biology (del Bigio, 2010). Finally, we also noted 1/30 GO-terms in each dataset relating to transport. This is consistent with the knowledge that these cells regulate the bidirectional transport of factors between CSF and interstitial fluid (Trillo-Contreras et al., 2019). There were also unique GO-terms relating to each dataset, but these were minimal and mostly did not have a common theme in comparison to those that were shared (5/30, 5/30, 1/30 unique GO-terms in *mouse* neonatal, *mouse* adult, and *human* adult datasets, respectively).

Given the striking conservation of ependymal cell GO-terms across age and species, we were interested in assessing whether this conservation was maintained across ependymal cell subtypes. To assess this, we sourced a single cell transcriptomic dataset from the *mouse* spinal cord, which is composed of E2 ependymal cells (Alfaro-Cervello et al., 2012). As with the forebrain datasets, clustering analysis was performed on spinal cord tissue in order to identify any cluster expressing genes that were specific to mature ependymal cells in the spinal cord. Indeed, we found one cluster expressing *Foxj1*, *Pifo*, and *Dynlrb1* (Figure 4A). This ependymal cell cluster was then compared to all other cell types in this dataset to generate the top 200 differentially expressed ependymal cell genes (for all genes significantly upregulated, *p* < 0.001 following Bonferroni correction, see Supplementary Table 1) then clusterProfiler was used to generate the top 30 most significant GO-terms (Figure 4B, Supplementary Figure 1D). As with forebrain ependymal cells, GO-terms are clustered into several major categories, including cilia-related terms and metal ion-related terms. Strikingly, GO-terms associated with metal ion-related functions made up 8/30 of the GO-terms demonstrating conservation of this *critically* understudied function, even in E2 ependymal cells. Not surprisingly 9/30 GO-terms were related to cilia consistent with the field focusing largely on this aspect of ependymal cell biology (del Bigio, 2010) but no transport-related GO-terms were detected. There were also unique GO-terms which made up far more of the top 30 GO-terms in the spinal cord (13/30) compared to the forebrain datasets which may suggest a more diverse repertoire of functions in E2 ependymal cells.

We performed validation experiments to confirm metal ion-related findings using RNAscope technology (Wang et al., 2012) on tissue sections collected from the brains of neonatal mice (*n* = 3 per group), postnatal week 12 adult mice (*n* = 3 per group), and adult human autopsy (*n* = 1), as well as from the spinal cords of postnatal week 12 adult mice (*n* = 3 per group). A primary gene involved in metal ion buffering is metallothionein 3 (*Mt3*) and we found that this gene was highly expressed in all ventricular and canal linings (74.6–100%, Figures 4C–H). Interestingly, *Mt3* was usually localized along the entire lining of the ventricles and the canal, including in the neonatal brain along the lateral border where mature ependymal cells do not reside. This suggests that metal ion-related functions in the ventricular lining may be initiated even before the full maturation of ependymal cells.

### Unique Ependymal Cell Signatures Across Age and Region

Albeit our analysis suggests a striking maintenance of ependymal cell function across diverse contexts, we were interested in further exploring potential differences between ependymal cells across age and region. To do this, we integrated *mouse* datasets from the neonatal brain, adult brain, and spinal cord in order to perform direct comparisons. Interestingly, the most striking difference between datasets existed when contrasting the spinal cord with brain datasets (Figure 5A) consistent with the spinal cord having more unique GO-terms (Figure 4B) and these cells being E2 cells in contrast to the E1 cells found in the neonatal and adult brain (Redmond et al., 2019). A top differentially expressed gene upregulated in spinal cord ependymal cells was *Plp1* (Figure 5A) consistent with a unique spinal cord GO-term: “oligodendrocyte differentiation” (Figure 4B). While neonatal and adult brain ependymal cells shared more similarities, there were some upregulated genes unique to the neonatal brain (including *Ccdc104* and *Ccdc19*, involved in the development of cilia) and some unique to the adult brain (including *Rarres2*, involved in initiating inflammation; Figure 5A).

We performed validation experiments on *Rarres2* to confirm its unique expression in the adult brain at the exclusion of the neonatal brain or spinal cord. This gene was chosen given its involvement in the inflammatory response and the topographic distribution of inflammatory lesions that appear to be highly prevalent in the parenchyma lining the ventricular system in the adult brain (Hatrock et al., 2020). We used RNAscope technology (Wang et al., 2012) on tissue sections collected from neonatal mice, postnatal week 12 adult mice, and spinal cord tissue (*n* = 3 per group; Figures 5B–E). Consistent with our transcriptomic analysis, we found that *Rarres2* was highly expressed in adult brain (100%), minimally expressed in the neonatal brain (12.28% ± 1.31%), and undetectable in the spinal cord (0%).

## Discussion

Our functional genomics analysis has confirmed a prominent role for cilia-related functions in ependymal cells consistent with the field (del Bigio, 2010; Wallmeier et al., 2019; Lorencova et al., 2020), which was apparent across age, species, and ependymal subtypes. In addition, we have uncovered an underappreciated function for ependymal cells: metal ion regulation, which is remarkably overrepresented, and conserved across species, age, and ependymal subtype. Although largely homogenous, ependymal cells across age and region also have some unique predicted functions as well. Together, our data proposes several underappreciated shared and unique ependymal functions that warrant further investigation.

Our analysis demonstrated that the top 30 GO-terms included four to nine metal ion-related terms across age, species, and ependymal subtypes. Albeit the adult mouse brain dataset revealed the most numerous metal ion-related GO-terms (nine out of the top 30 terms were metal ion-related), these terms were also well represented in the adult human brain and spinal cord (8 out of 30 terms), which suggests a critical role for metal ion regulation in the maintenance of adult brain homeostasis irrelevant of ependymal subtype. Metal ion regulation is critical for the maintenance of homeostasis throughout the body, which, leads to oxidative stress and toxicity, if dysregulated (Valko et al., 2005; Salim, 2017). Indeed, several diseases such as cancer (Valko et al., 2006), autism (Nadeem et al., 2020), Wilson’s disease (Kitzberger et al., 2005), and Alzheimer’s disease (Lovell et al., 1998; Miller et al., 2006; Everett et al., 2021) are associated with a dysregulation of metal ion homeostasis. Very few studies have analyzed the role of metal ion regulation in the brain, nor how ependymal cells might be involved. In 2008, Zatta et al. (2008) investigated metal ion accumulation in the brain of aging cattle and found that the accumulation of copper, zinc, and manganese was dependent on age. They also found that the major cell types expressing metallothionein, which is the major metal-ion binding protein that buffers heavy metals, were ependymal cells and astrocytes. In our analysis, we also identified metallothionein in both ependymal cells and astrocytes, although it was only ependymal cells, not astrocytes, that were enriched in GO-terms associated with metal ion regulation (astrocyte data not shown), which could suggest a more prominent role for ependymal cells in regulating metal ion homeostasis. There are three major metallothioneins. MT1 and MT2 are found non-specifically across mammalian organ systems, while MT3 is predominantly CNS-specific (Coyle et al., 2002; Miyazaki et al., 2002). Interestingly, MT3 is downregulated in Alzheimer’s brains (El Ghazi et al., 2006) but how this contributes to disease pathogenesis and whether a dyshomeostasis in ependymal cell-specific metal ion detoxification function contributes to disease is unknown (Everett et al., 2021).

Interestingly, neonatal ependymal cells had the least number of metal ion GO-terms (4 out of the top 30 terms). Rather these ependymal cells are dominated by GO-terms associated with ciliogenesis and cilia motility, consistent with these cells developing cilia at this early-stage of life (Jacquet et al., 2009). The presence of other GO-terms associated with developmental processes such as determination of left/right symmetry also appeared in neonatal ependymal cells. This is consistent with ependymal cells playing a role in determining the symmetrical development of mammals across the medio/lateral plane (Chen et al., 1998; Wallmeier et al., 2019). Indeed, the absence of functional genes in ciliopathies such as in primary ciliary dyskinesia (PCD) and Kartagener syndrome, can lead to asymmetrical development. For instance, in *FOXJ1* mutant patients, left/right asymmetry occurs and leads to organ laterality defects (Catana and Apostu, 2017). It is thought that during embryogenesis, when ciliary beating patterns are disrupted, the laterality of organ placement is disrupted, and in the most severe cases can result in long-term defects including congenital heart defects. The contribution of ependymal cells in maintaining body symmetry during development has not been studied.

We noted the presence of immune-related genes, such as retinoic acid receptor responder protein 2 (*RARRES2* or Chemerin) which was largely restricted to adult ventricular ependymal cells. *RARRES2* is a 14 kDa protein secreted in an inactive form (prochemerin) which is then activated through the cleavage of the C-terminus by inflammatory serine proteases (Zabel et al., 2005). It is a potent chemoattractant that regulates the recruitment of circulating immune cells *via* binding of the G protein-coupled receptor CMKLR1 (Wittamer et al., 2003; Zabel et al., 2008; Huang et al., 2020). Interestingly, in multiple sclerosis—a common chronic inflammatory disease of the CNS, the most consistent region of inflammatory lesion formation is the periventricular area, directly adjacent to ependymal cells lining the brain’s ventricles (Haider et al., 2016). Why immune cells target this defined region is not known. While lesions also form in the spinal cord, these lesions are not as commonly found lining the spinal canal, which raises the possibility that ependymal cells may play a critical role in the recruitment of immune cells in the brain *via* chemoattractants such as *RARRES2*.

Based on our GO-term analysis, the presence of genes responsible for transport functions in ependymal cells from both the human and mouse ventricles suggests that E1 cells play a critical transport role, which appears to be less prominent in E2 cells from the spinal cord. Aquaporin-4, which is a membrane channel protein, is one transport-associated gene driving the extracellular transport GO-term in our forebrain datasets. Aquaporin-4 responds passively to osmotic gradients and is responsible for the fast transportation of water through its cell membrane. This is critical for the efficient operation of the glymphatic system, important for waste removal, as well as fine-tuning potassium homeostasis. The mechanisms of glymphatic waste clearance in the CNS have been studied extensively in the brain but not the spinal cord (Dupont et al., 2019). These findings suggest glymphatic clearance may have unique regulatory mechanisms in the spinal cord compared to the brain, warranting further investigation.

E2 cells have previously been shown to be capable of contributing to the regenerative response following spinal cord injury (Johansson et al., 1999; Namiki and Tator, 1999; Mothe and Tator, 2005), consistent with spinal cord ependymal cells in our dataset being associated with a GO-term related to the regulation of precursor cell proliferation (“negative regulation of neural precursor cell proliferation”). This GO-term was not detected in brain ependymal cells suggesting that E2 cells may be uniquely involved in actively suppressing proliferative responses in normal health, but under disease or injury conditions, this suppression may be lifted unleashing a dormant regenerative capacity. Interestingly, our previous data assessing the regenerative potential of ependymal cells in the lateral ventricles of the adult mouse did not detect any regenerative potential of E1 cells (Shah et al., 2018). Further studies are needed to clarify the functional similarities and differences between E1 and E2 ependymal cells in relation to their regenerative potential.

Our data implicate ependymal cells in several under-characterized functions related to maintaining brain homeostasis throughout life. Most immediately, deciphering the role of ependymal-mediated metal ion regulation will be critical to understanding the contribution of these cells to regulating oxidative stress and toxicity in health and disease.

## Data Availability Statement

The original contributions presented in the study are included in the article/Supplementary Material, further inquiries can be directed to the corresponding author.

## Ethics Statement

The animal study was reviewed and approved by McGill University Animal Care and Use Committee.

## Author Contributions

AM carried out most bioinformatic and RNAscope work. BL, DH, and NC-D supported tissue processing and RNAscope work. MC supported bioinformatics work, while GB supervised MC. AM prepared figures and drafted the manuscript. JS conceived and designed the project, supervised the work, and prepared and approved the final manuscript. KP provided human single cell data. All authors contributed to the article and approved the submitted version.

## Conflict of Interest

The authors declare that the research was conducted in the absence of any commercial or financial relationships that could be construed as a potential conflict of interest.

## Footnotes

^1^https://github.com/StrattonLab/scRNAseq_Frontiers

## Supplementary Material

SUPPLEMENTARY FIGURE 1 GO-analysis networks displaying gene-GO-term connections for each ependymal cell cluster. Top 30 GO-terms (tan nodes) for each ependymal cluster are displayed, demonstrating the genes (gray nodes) that are associated with GO-terms. All included genes are part of the top 200 upregulated genes for that ependymal cluster. Ependymal clusters are displayed for neonatal mouse brain **(A)**, adult mouse brain **(B)**, adult human brain **(C)**, and mouse spinal cord **(D)**.
