# Supplementary figures and images for: Single Cell Transcriptomics of Ependymal Cells Across Age, Region and Species Reveals Cilia-Related and Metal Ion Regulatory Roles as Major Conserved Ependymal Cell Functions
Source: Front Cell Neurosci. 2021 Jul 15;15:703951. doi: 10.3389/fncel.2021.703951 (PMC8319996; doi:10.3389/fncel.2021.703951)

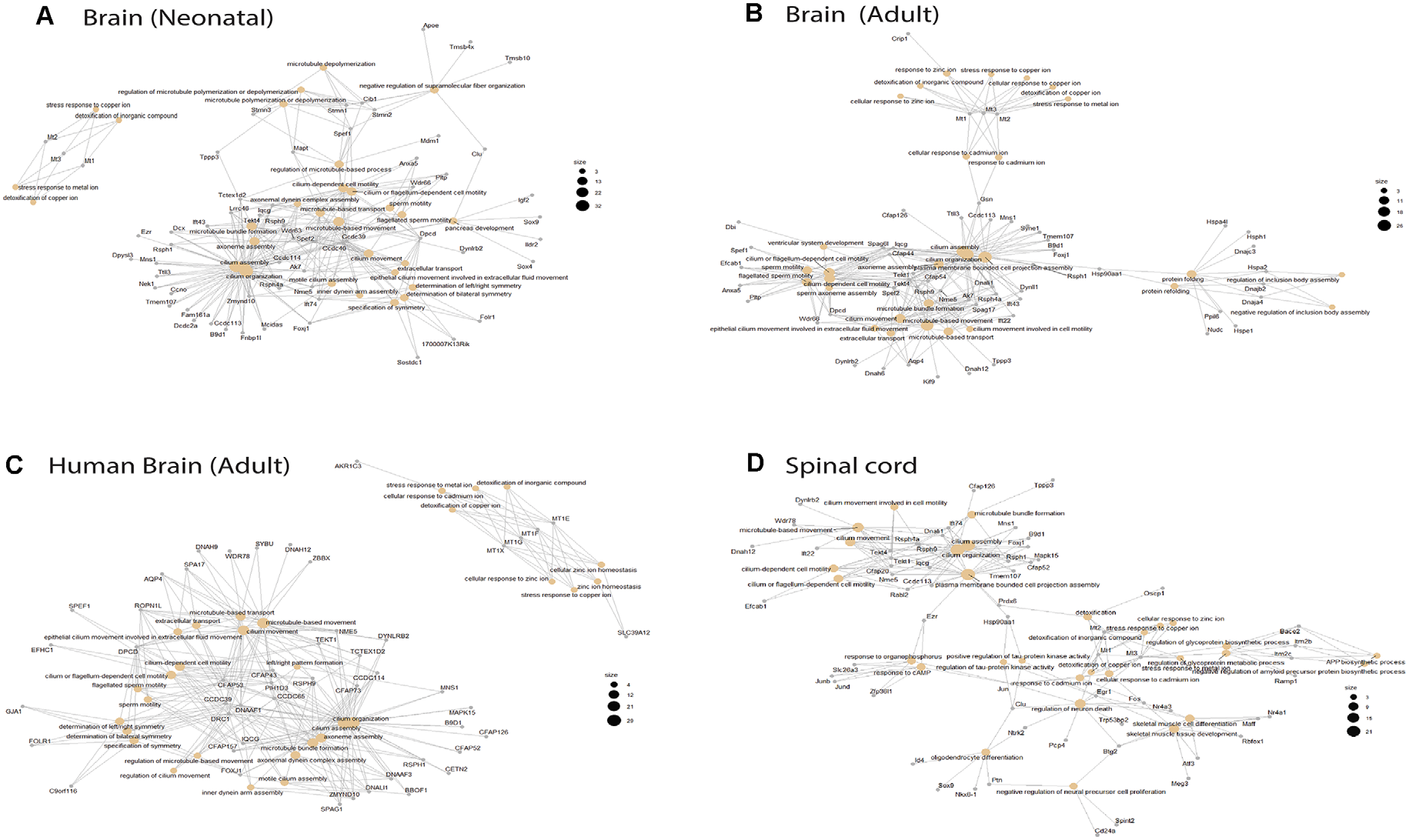

Supplement: SUPPLEMENTARY FIGURE 1 — GO-analysis networks displaying gene-GO-term connections for each ependymal cell cluster. Top 30 GO-terms (tan nodes) for each ependymal cluster are displayed, demonstrating the genes (gray nodes) that are associated with GO-terms. All included genes are part of the top 200 upregulated genes for that ependymal cluster. Ependymal clusters are displayed for neonatal mouse brain (A), adult mouse brain (B), adult human brain (C), and mouse spinal cord (D). [file Image_1.tif]
